# Functional analysis of the role of hydrogen sulfide in the regulation of dark-induced leaf senescence in Arabidopsis

**DOI:** 10.1038/s41598-017-02872-0

**Published:** 2017-06-01

**Authors:** Bo Wei, Wei Zhang, Jin Chao, Tianru Zhang, Tingting Zhao, Graham Noctor, Yongsheng Liu, Yi Han

**Affiliations:** 1 School of Food Science and Engineering, Hefei University of Technology, Hefei, Anhui 230009 China; 2 Institute of Plant Sciences Paris Saclay, Université Paris-Sud, Centre National de la Recherche Scientifique, Institut National de la Recherche Agronomique, Université Evry, Paris Diderot, Sorbonne Paris-Cité, Université Paris-Saclay, 91405 Orsay, France

## Abstract

There is growing evidence that hydrogen sulfide (H_2_S) is involved in many physiological processes in plants, but the role of H_2_S in dark-induced leaf senescence remains unknown. In this work, we found that H_2_S not only inhibited chlorophyll degradation but also caused the accumulation of photoreactive pheide *a* in detached leaves under extended darkness. Despite this, transcript levels of *senescence-associated genes* (*SAGs*) were less affected in H_2_S-treated detached leaves compared with those in H_2_S-untreated detached leaves. Furthermore, cell death/rapid bleaching occurred in both H_2_S-treated detached and attached leaves after transfer from extended darkness to light. Unlike the lack of effect of H_2_S on *SAG* transcripts in darkened detached leaves, exogenous H_2_S induced higher SAG transcript levels in attached leaves than untreated attached leaves. Genetic evidence further underlined the positive correlation between SAG expression in attached leaves and H_2_S. In addition, effects of H_2_S on SAG expression in attached leaves were compromised in the *S*-nitrosoglutathione reductase-deficient mutant, *gsnor1*. Taken together, our results suggest that H_2_S suppresses chlorophyll degradation of detached leaves by regulating a dark-dependent reaction, and that this gas positively modulates SAG expression in attached leaves under prolonged darkness in a GSNOR1-dependent manner.

## Introduction

Hydrogen sulfide (H_2_S) is a pungent colorless gas with a distinctive rotten-egg odor, often regarded as an environmental pollutant and a toxin for almost all organisms. One of the well-known mechanisms for H_2_S toxicity involves inhibition of one of the key enzymes in the mitochondrial respiratory chain, cytochrome *c* oxidase^1, 2^. Despite the toxicity of H_2_S, it is well established that plants can themselves generate and release this gas, especially when exposed to external cysteine, sulfate, sulfite or SO_2_^3–5^. This is thought to be a mechanism for dissipation of excess sulfur^6^, but certain adverse environmental stimuli such as pathogens and drought can also stimulate H_2_S emissions above basal, endogenously produced rates^7, 8^.

Plants can produce H_2_S through sulfite reductase, which catalyzes the reduction of sulfite to sulfide, or through two cysteine-dependent reactions involving members of the *O*-acetylserine(thiol)lyase (OAS-TL) gene family. L-cysteine desulfhydrase (DES, EC 4.4.1.1) converts L-cysteine to H_2_S, ammonia and pyruvate^9^ while β-cyanoalanine synthase produces H_2_S via detoxification of cyanide at the expense of cysteine^10, 11^. Another potential enzyme in plant H_2_S homeostasis is a D-cysteine desulfhydrase, which, similar to DES, produces H_2_S, ammonia and pyruvate^12^. However, the physiological function of D-cysteine desulfhydrase is completely unknown.

Many studies published since the late 1990s have shown that H_2_S can have signalling, defense and anti-apoptotic functions in mammalian systems^13^. The discovery of these novel functions of H_2_S in mammals stimulated work in plants, leading to an appreciation of the important and varied physiological functions of H_2_S^6, 13–18^. This gas has not only been implicated in seed germination, root development, and photosynthesis^19–28^, but can also enhance plant acclimation/tolerance to various stresses such as drought, heavy metals, salinity, cold, heat and osmotic stress^8, 29–36^. One notion is that the influence of H_2_S on stress responses is at least partly linked to enhanced antioxidant capacity^6^. Consistent with this notion, it has very recently been reported that several antioxidant components such as cytosolic ascorbate peroxidase1, 2-Cys peroxiredoxin A or B, and peroxisomal catalase3 in Arabidopsis plants, underwent *S*-sulfhydration in the presence of exogenous H_2_S, leading to enhanced enzyme activities^37^. H_2_S is also the end-product of assimilatory sulfate reduction, in which it is incorporated into OAS-TL to produce cysteine, the source of reduced sulphur including the redox buffer, glutathione^38^. Indeed, prolonged treatment with H_2_S leads to increased glutathione synthesis. This effect may have a direct protective effect during stress or, alternatively, may act to regulate defense genes that play important roles in adverse environmental conditions such as cadmium exposure or drought^39–43^. Thus, there are several mechanisms by which H_2_S could act to regulate stress responses by affecting antioxidant status, but their relative importance is still uncertain.

Apart from possible effects mediated by H_2_S modulation of cell thiol status, recent work has shown that this gas can have a signalling function through other pathways. For instance, genetic evidence revealed that DES1 deficiency leads to the accumulation and lipidation of ATG8 isoforms in Arabidopsis, which is associated with autophagy activation. Exogenous sulfide suppresses autophagy induction in Arabidopsis *des1* mutants under nutrient-rich conditions and in wild type plants under nitrogen deprivation, whereas glutathione had no effect^44^. Interestingly, sulfide did not scavenge reactive oxygen species (ROS) triggered by nitrogen starvation, in contrast to glutathione. These results indicate that sulfide represses autophagy via mechanisms that are independent of redox conditions^44, 45^. However, Scuffi *et al*. (2014) found that the lack of cytosolic H_2_S in *des1* significantly decreases endogenous nitric oxide (NO) levels and that NO acts downstream of H_2_S to close stomata via an ABA-dependent pathway^26^. These observations draw attention to the potential importance of H_2_S and its interactions with NO status in regulating various biological processes in plants. However, the signal mechanisms and direct downstream targets of H_2_S that regulate stomatal movement and autophagy remain to be identified.

The overall aim of the present work was to investigate the significance of H_2_S in modulation of processes involved in dark-induced senescence in plants. The specific objectives were (1) to assess the effect of H_2_S on dark-induced chlorophyll loss; (2) to establish whether H_2_S affects chlorophyll loss via alterations in autophagy and well-characterized senescence pathways; (3) to investigate the links between H_2_S and chlorophyll breakdown intermediates that are known to be implicated in cell death; and (4) to evaluate the role of cell redox components in mediating the effect of H_2_S. The results show that H_2_S favors a stay-green phenotype in detached leaves by affecting a dark-dependent reaction involved in chlorophyll degradation and that this gas regulates SAG expression in attached leaves through processes linked to NO homeostasis.

## Results

### Effect of H_2_S on dark-triggered leaf chlorophyll degradation in detached leaves

**Figure 1:**
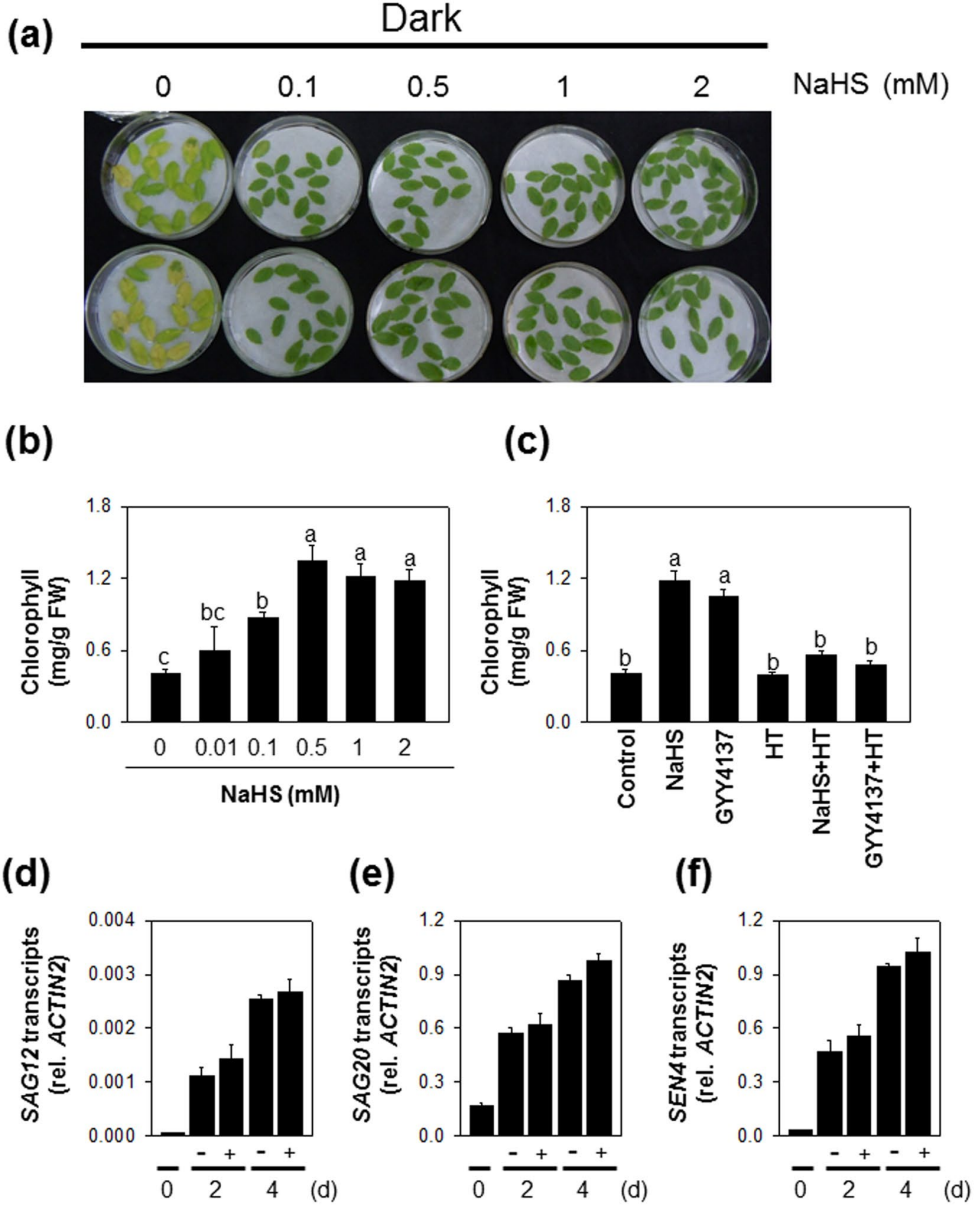
Effect of H_2_S exposure on chlorophyll breakdown and SAG expression in detached leaves during extended darkness for up to 4 d. (**a**,**b**), effects of H_2_S gas released from 0 to 2 mM H_2_S donor NaHS solution (see Materials and Methods) on leaf yellowing and chlorophyll content, respectively, at 4 d of darkness. Effect of another H_2_S donor GYY4137 and H_2_S scavenger HT (**c**) on chlorophyll degradation under extended darkness for 4 d. For GYY4137 and HT treatments, 3-week-old detached leaves were floated in petri dishes containing 3 mL solution of 0.1 mM GYY4137 alone, 0.1 mM HT alone or 0.1 mM GYY4137 plus 0.1 mM HT combined treatment. Transcript levels of *SAG12* (**d**), *SAG20* (**e**) and *SEN4* (**f**) in detached leaves of wild type subjected to H_2_S or H_2_S-free treatment for up to 4 d of complete darkness. + and − indicate detached leaves of Col-0 fumigated with or without 0.5 mM NaHS respectively. Data are means ± SE of at least three independent samples from different plants. Letters indicates significant difference from the wild type at *P* < 0.05, using the Student’s *t* test.

Prolonged darkness is often used to induce rapid and synchronous senescence in detached leaves. Hence, a dark-detached system has been widely used as a model to study senescence-associated regulatory mechanisms. Loss of chlorophyll has often been exploited as a well-characterized marker of dark-induced leaf senescence. To investigate the potential role of H_2_S in leaf chlorophyll metabolism, detached leaves were fumigated with H_2_S, released from 0.01 to 2 mM NaHS solution (see Materials and Methods), and chlorophyll content was assessed after extended darkness for 4d. Under normal growth conditions, leaf chlorophyll level was about 1.37 mg/g fresh weight. Extended darkness led to a loss of leaf color and a corresponding decrease in chlorophyll level in excised leaves of the wild type examined without treatment with H_2_S (Fig. 1a,b). In contrast, treatment with NaHS at external concentrations of 0.01, 0.1, 0.5, 1.0 and 2.0 mM significantly suppressed chlorophyll loss in a dose-dependent manner (Fig. 1b). Thus, H_2_S treatment caused a “stay-green” phenotype.

To confirm the effect of H_2_S on chlorophyll degradation, another H_2_S donor (GYY4137) and a H_2_S scavenger (hypotaurine; HT) were employed. Consistently, it was found that H_2_S generated from 100 µM GYY4137 had the same effect on leaf chlorophyll content as NaHS treatment. In contrast, HT completely blocked the effects of both NaHS and GYY4137 treatment (Fig. 1c). Together, these results suggest that H_2_S plays a negative role in chlorophyll degradation.

Links between H_2_S and autophagy have recently been reported, and many autophagy-deficient mutants display an early senescence phenotype under extended darkness^45–47^. We therefore examined whether autophagy might be involved in the regulation of chlorophyll degradation by H_2_S. Consistent with previous studies, detached leaves from 3-week-old autophagy deficient mutants *atg2* or *atg18a* kept in darkness for 2 days exhibited a much greater senescence-associated loss of green leaf color than those from the wild type (Supplementary Fig. S1a). However, leaf yellowing and chlorophyll degradation in the autophagy-deficient mutant were markedly suppressed in the presence of H_2_S at 2 or 4 d of extended darkness (Supplementary Fig. S1a–c). Furthermore, H_2_S contents and the activities of enzymes with H_2_S-releasing activity (LCD and DCD) were not decreased in the *atg* mutants compared with those in Col-0 (Supplementary Fig. S1d–f). These results suggest that the negative effect of H_2_S on chlorophyll loss under extended darkness is independent of the autophagic pathway.

H_2_S is the substrate for the biosynthesis of cysteine^2^, and cysteine and cysteine-containing compounds such as glutathione are key determinants of cell redox homeostasis^48, 49^. In a first step to study possible links between H_2_S and cellular thiols in the regulation of chlorophyll degradation, the effects of exogenous supply of either cysteine or glutathione on the loss of leaf color upon exposure to extended darkness were investigated. Unlike H_2_S, neither cysteine nor glutathione treatments affected the loss of leaf colour and chlorophyll degradation in Col-0 (Supplementary Fig. S2a). In a second approach, the importance of changes in endogenous cysteine and glutathione was examined in the *cad2* mutant, which has higher cysteine contents than Col-0^42^. In the absence of H_2_S, the *cad2* mutant showed similar dark-induced loss of chlorophyll to Col-0, while H_2_S treatment produced a similar stay-green effect in both genotypes (Supplementary Fig. S2b). On the other hand, no visible difference in either glutathione levels or H_2_O_2_ contents was observed between H_2_S-treated wild type and the control treatment (Supplementary Fig. S2c,d). Thus, these observations provide little evidence that the inhibitory effect of H_2_S on chlorophyll degradation under extended darkness is linked to increased cysteine and glutathione.

Many genes that are up-regulated during the senescence processes have been defined as senescence-associated genes (SAGs). These notably include *SAG12*, *SAG20* and *SEN4* ^50^. The transcript levels of these *SAGs* were determined in the wild type in the presence or absence of H_2_S treatment. Intriguingly, dark treatment led to significant increases in transcript levels of *SAG12*, *SAG20* and *SEN4* in wild type with or without H_2_S treatment (Fig. 1d–f). Thus, the expression patterns of SAGs are not correlated with an inhibition of chlorophyll metabolism in H_2_S-treated detached leaves under extended darkness. From this observation, we conclude that the stay-green effects of H_2_S are not occurring by a general modulation of dark-induced senescence pathways.

### Effect of H_2_S on chlorophyll breakdown intermediates and cell death in detached leaves

**Figure 2:**
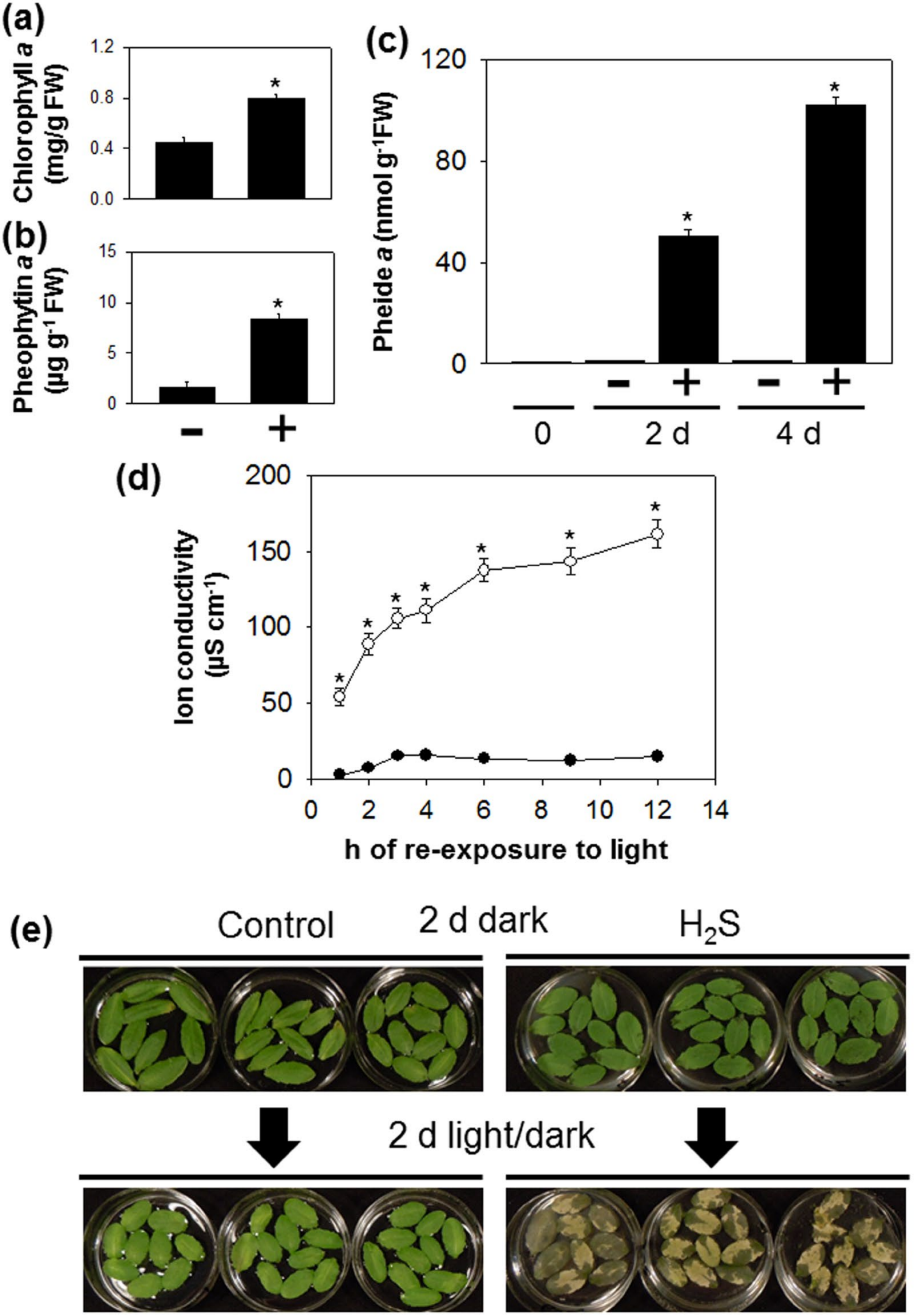
Effects of H_2_S exposure on accumulation of green intermediates of chlorophyll breakdown and cell death in detached leaves. Amounts of chlorophyll *a* (**a**) and pheophytin *a* (**b**) accumulation in detached leaves of wild type subjected to H_2_S or H_2_S-free treatment for up to 4 d of complete darkness. (**c**), Accumulation of pheide *a* in response to dark incubation of detached leaves of control and H_2_S-treated wild type for up to 4 d. + and − indicate detached leaves of Col-0 fumigated with or without 0.5 mM NaHS, respectively. (**d**), Determination of ion leakage as a measure for cell death in detached leaves treated with H_2_S (white bars) or without H_2_S (black bars). Before re-exposure to the light for up to 12 h, detached leaves were incubated in the presence or absence of 0.5 mM NaHS in the darkness for 2 d. (**e**), Photographs of detached wild type leaves treated with H_2_S released from 0.5 mM NaHS solution under extended darkness for 2 d and transfer to regular growth conditions for another 2 d. Data are means ± SE of at least three independent samples from different plants. Asterisks indicate significant difference between H_2_S-treated wild type and control wild type at the same time point at *P* < 0.05, using the Student’s *t* test.

To gain insight into the role of H_2_S in the regulation of chlorophyll degradation, green pigments were extracted and separated by HPLC. After 4 d of complete darkness, levels of chlorophyll *a* decreased faster in the control wild type than in H_2_S-treated plants (Fig. 2a). Moreover, H_2_S-treated plants accumulated increasing amounts of pheophytin *a* under 4 d of extended darkness (Fig. 2b) HPLC analyses also showed that the basal level of pheide *a* was very low (around 0.95 nmol.g^−1^ FW before dark treatment) and did not increase appreciably in detached leaves kept in the dark on water (Fig. 2c). In contrast, H_2_S treatment resulted in the accumulation of pheide *a* in a time-dependent manner (Fig. 2c) that correlated well with the “stay-green” phenotype (Fig. 1a).

Conversion of pheide *a* to red chlorophyll catabolite (RCC) by pheophorbide *a* oxygenase (PAO) is the critical step of loss of green pigment^51, 52^. Quantification of transcripts for enzymes involved in chlorophyll breakdown provided little evidence that H_2_S-mediated regulation of pheide *a* contents occurs at the transcriptional level (Supplementary Fig. S4). While *PAO* transcript abundance increased during darkness, this response was not significantly affected by H_2_S treatment. Transcript levels of other chlorophyll catabolic genes^51, 53–56^ were also similar in detached leaves treated or not with H_2_S during extended darkness (Supplementary Fig. S4). Exceptions were transcripts for *CLH2* (*CHLOROPHYLLASE 2*) and *NYC1* (*NON-YELLOW COLORING 1*), which were increased in H_2_S-treated leaves compared to controls at 2 d of darkness (Supplementary Fig. S4).

Light-dependent cell death could be induced by pheide *a* ^52^. While the water control showed very low ion leakage upon exposure to light, the stay-green phenotype associated with H_2_S treatment was accompanied by higher ion conductivity (Fig. 2d). Interestingly, ion leakage increased substantially on re-illumination whereas it stayed low in the water-treated controls (Fig. 2d). Moreover, on transfer to a standard light/dark regime, H_2_S-treatment induced a visible cell death phenotype that was not observed in water-treated controls or in H_2_S-treated detached leaves under standard growth conditions (16 h light/8 h dark; Fig. 2e; Supplementary Fig. S3). These results provide additional evidence that H_2_S-indcued suppression of chlorophyll degradation in detached leaves under extended darkness is unlikely to occur by regulation of known senescence-associated pathways.

### Effect of H_2_S on phenotype and SAG expression in attached leaves subjected to extended darkness

**Figure 3:**
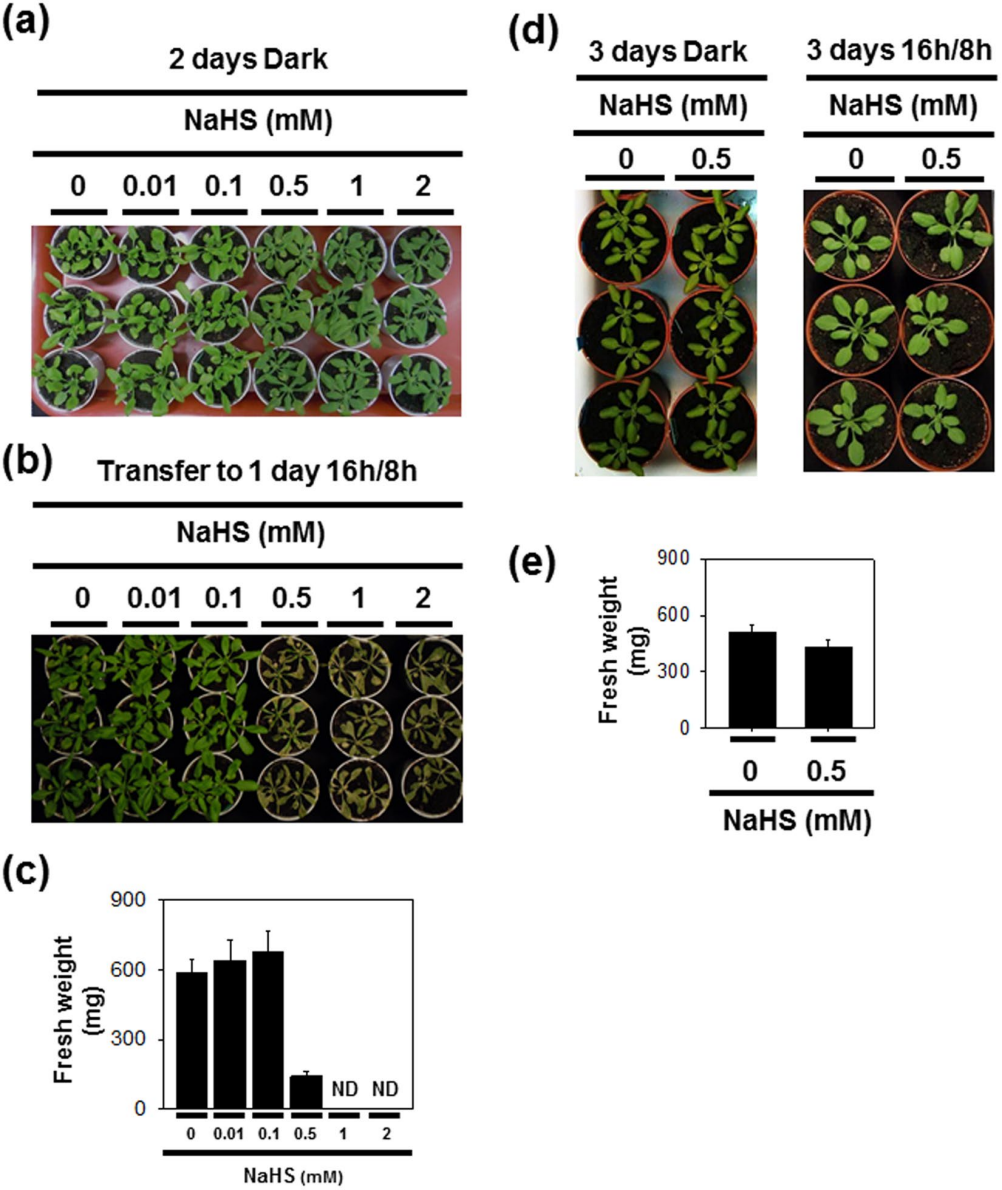
Effects of H_2_S exposure on phenotype of whole plants under prolonged darkness and regular growth conditions. Photographs of whole plants treated with different NaHS concentrations exposed to complete darkness for 2 d (**a**) and then transfer to regular growth conditions for another 1 d (**b**) (16 h light/8 h dark photoperiod). The fresh weight of plants treated with the indicated concentrations of NaHS was measured 6 d after transfer from continuous darkness to light-dark conditions (**c**). (**d**), Effects of 0.5 mM NaHS treatment under prolonged darkness or regular 16 h light/8 h dark conditions for 3 days. (**e**) Fresh weight were taken at 7 d after H_2_S treatment under 16 h light/8 h dark conditions. Data are means ± SE of at least 15 different plants. ND: not detected.

H_2_S and dark treatments were performed on attached leaves. Neither the visual phenotype nor the chlorophyll contents of plants treated with different concentrations of NaHS was distinguishable from the untreated control after 2 d darkness (Fig. 3a; Supplementary Fig. S5a). However, within 1 d after transfer to the light/dark regime following exposure to 2 d of darkness, intact plants treated with NaHS at a concentration of 0.5 mM or above exhibited rapid loss of green pigments, including pheophytin *a* (Fig. 3b,c; Supplementary Fig. S5b). This effect is very similar to that observed in H_2_S-treated detached leaves after transfer from extended darkness to light (Fig. 2b). In contrast, effects of H_2_S on intact plants under complete darkness and regular light/dark cycles were less evident (Fig. 3d,e; Supplementary Fig. S5c,d). These data demonstrate that H_2_S triggers a phenotype of bleaching or cell death in both attached and detached leaves shifted from continuous darkness to light through one or more processes that require light.

**Figure 4:**
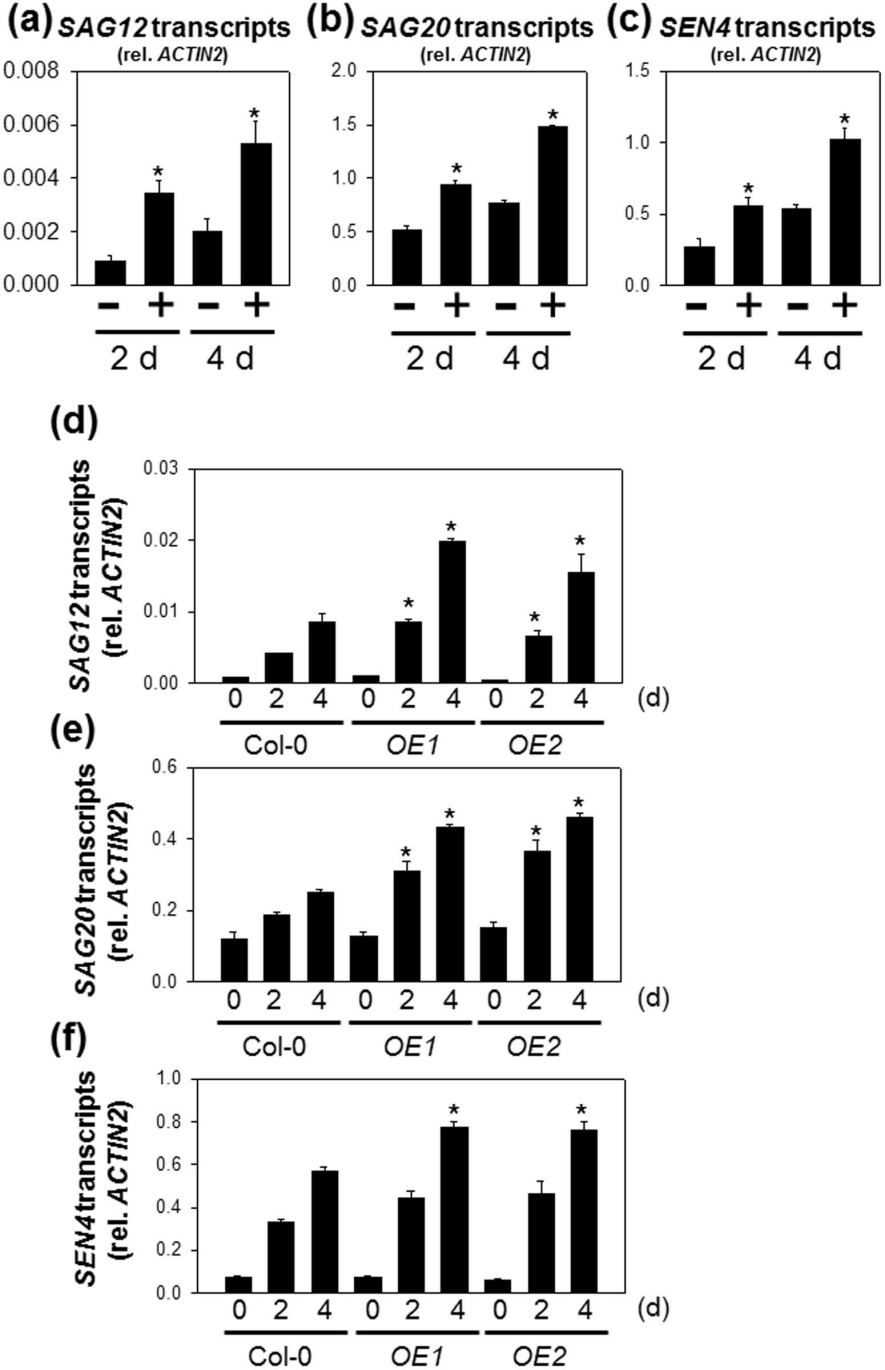
Effects of exogenously applied H_2_S and *DES1* transgenic lines on SAG expression in attached leaves under extended darkness. Transcript levels of *SAG12* (**a**), *SAG20* (**b**) and *SEN4* (**c**) in wild-type plants subjected to 0.5 mM NaHS treatment plus complete darkness. Samples were taken from the attached leaves at 2 or 4 d of darkness. + and − indicate intact plants fumigated with or without 0.5 mM NaHS, respectively. (**d**), *SAG12*. (**e**), *SAG20*. (**f**), *SEN4*. Samples were taken from the attached leaves at 2 and 4 d of darkness. *OE1* and *OE2* indicate two independent *DES1* overexpression lines. Data are means ± SE of at least three independent samples from different plants. Asterisks indicate significant difference from the wild type at the same time point at *P* < 0.05, using the Student’s *t* test.

To investigate the influence of H_2_S on dark-induced senescence processes in attached leaves at the transcriptional level, expression analyses of *SAG12 SAG20* and *SEN4* were performed. As shown in Fig. 4a–c, H_2_S-treatment significantly increased expression of these genes in plants placed in darkness. In contrast, H_2_S treatment did not affect their transcript levels in plants in the repeated light/dark conditions at 2 d or 4 d (Supplementary Fig. S6). To directly test if endogenous H_2_S produces a similar effect on the expression of SAGs, transgenic plants expressing *DES1* were generated. Two independent *DES1* OE lines showed significant increases in both leaf total LCD activity and intracellular H_2_S contents (Supplementary Fig. S7a–c). As described above for exogenous H_2_S, *DES1*-dependent increases in leaf H_2_S significantly stimulated accumulation of *SAG12*, *SAG20* and *SEN4* transcripts above Col-0 values, when plants were kept in darkness for 2d or 4d (Fig. 4d–f).

**Figure 5:**
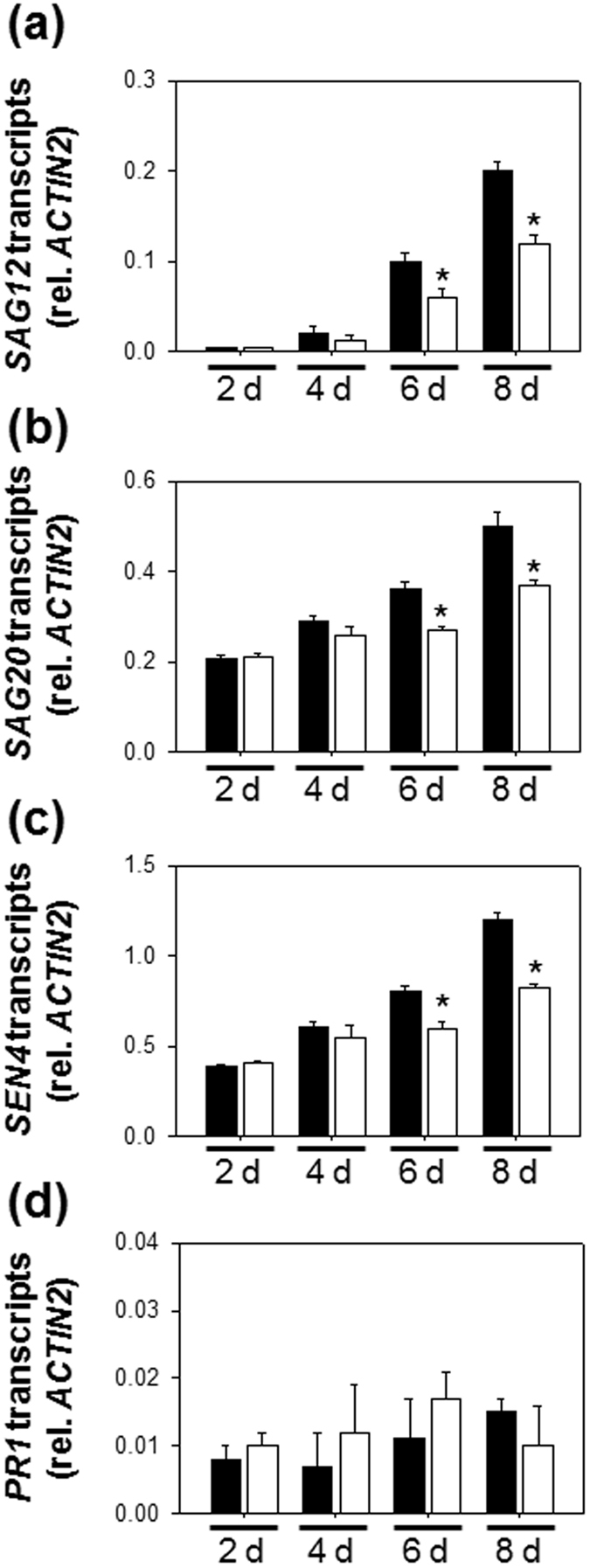
Expressions of of *SAGs* and *PR1* gene in attached leaves of Col-0 and *des1* mutant during extended darkness. (**a**), *SAG12*. (**b**), *SAG20*. (**c**), *SEN4*. (**d**), *PR1*. Ten-day-old seedlings of Col-0 and *des1* were incubated under extended darkness for up to 8 d. White bars, *des1* mutant. Black bars, Col-0. Asterisks indicate significant difference from the wild type at the same time point at *P* < 0.05, using the Student’s *t* test.

To analyze the role of *DES1* further, we exploited the *des1* mutant, which is impaired in LCD expression and activity (Supplementary Fig. S7d,e), and in H_2_S generation (Fig. 5)^45^. During extended darkness, increases in *SAG* transcript levels were significantly higher in the wild type than in *des1* attached leaves (Fig. 5). In contrast to its effect on SAG expression, the *des1* mutation did not affect transcript levels for the salicylic acid (SA)-dependent gene, *PR1* (Fig. 5). Hence, evidence from both OE and loss-of-function lines (Figs 4 and 5) suggests that H_2_S is a regulator of SAG expression in attached leaves.

### Effect of H_2_S on oxidative stress and the ascorbate-glutathione pathway in attached leaves

**Figure 6:**
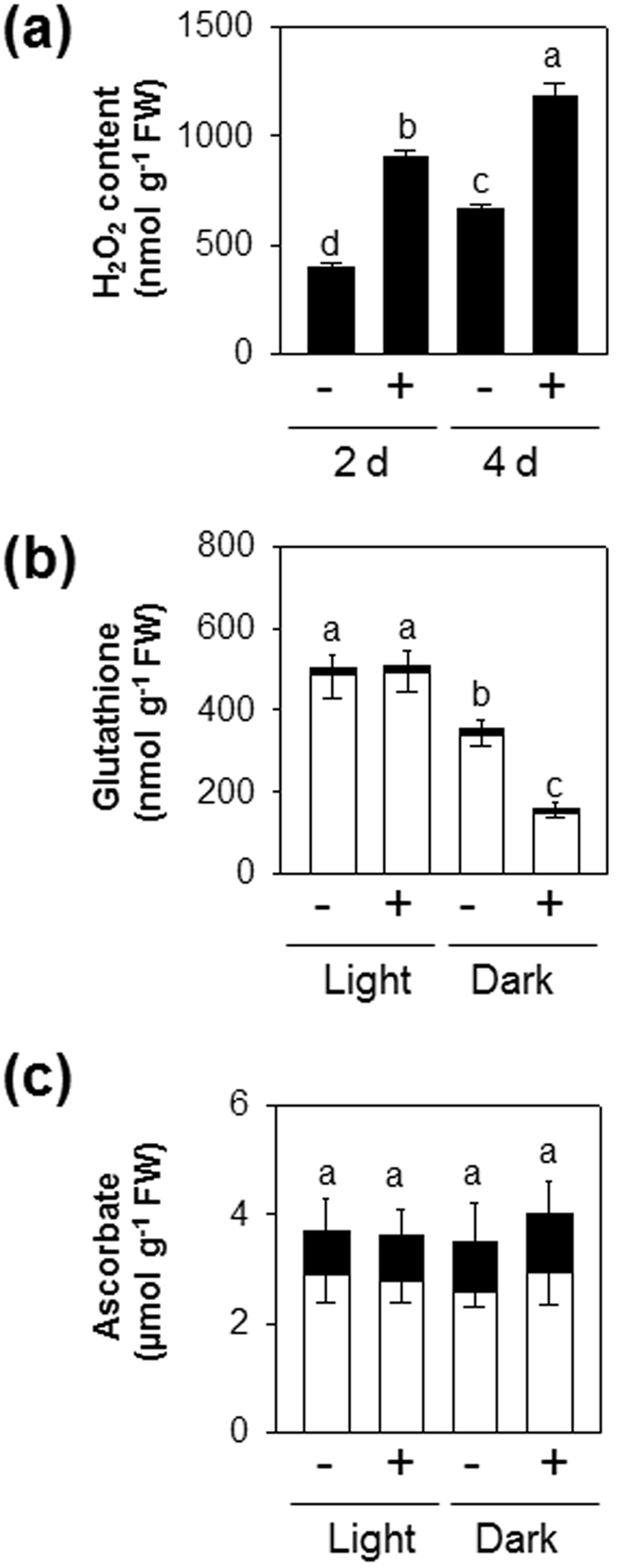
Effects of H_2_S exposure on leaf H_2_O_2_, glutathione and ascorbate in attached leaves of Col-0 under extended darkness and normal growth conditions. (**a**), H_2_O_2_ content. Samples were taken from the attached leaves at 2 and 4 d of darkness. (**b**), reduced glutathione (white bars) and oxidized glutathione (black bars). (**c**), ascorbate (white bars) and dehydroascorbate (black bars). Samples were taken from the attached leaves at 2 d of darkness and regular growth conditions within 16 h light/8 h dark photoperiod. + and − indicate intact plants fumigated with or without 0.5 mM NaHS, respectively. Light indicates regular growth conditions within 16 h light/8 h dark photoperiod. Data are means ± SE of at least three independent samples from different plants. Letters indicates significant difference from the wild type at *P* < 0.05, using the Student’s *t* test.

Leaf senescence-linked events are often associated with pronounced accumulation of ROS^47^. Thus, levels of hydrogen peroxide (H_2_O_2_) were monitored in H_2_S-treated attached leaves under dark incubation. Although no difference in H_2_O_2_ contents was observed between H_2_S-treated and and -untreated whole plants in normal growth conditions (Supplementary Fig. S8), dark-induced H_2_O_2_ generation was further enhanced by H_2_S treatment (Fig. 6a). Because the glutathione pool is in close correspondence to H_2_O_2_ availability^40, 57^, we analyzed this key intracellular thiol-disulfide compound. Treatment with NaHS for 2 d resulted in a dramatic decrease in the content of GSH (Fig. 6b). Like glutathione, ascorbate is an abundant and stable redox buffer required for H_2_O_2_ metabolism, and is considered to be partly regenerated by glutathione^48^. Despite this, the effects of H_2_S on glutathione pools were not associated with marked perturbation of leaf ascorbate pools (Fig. 6b,c).

**Figure 7:**
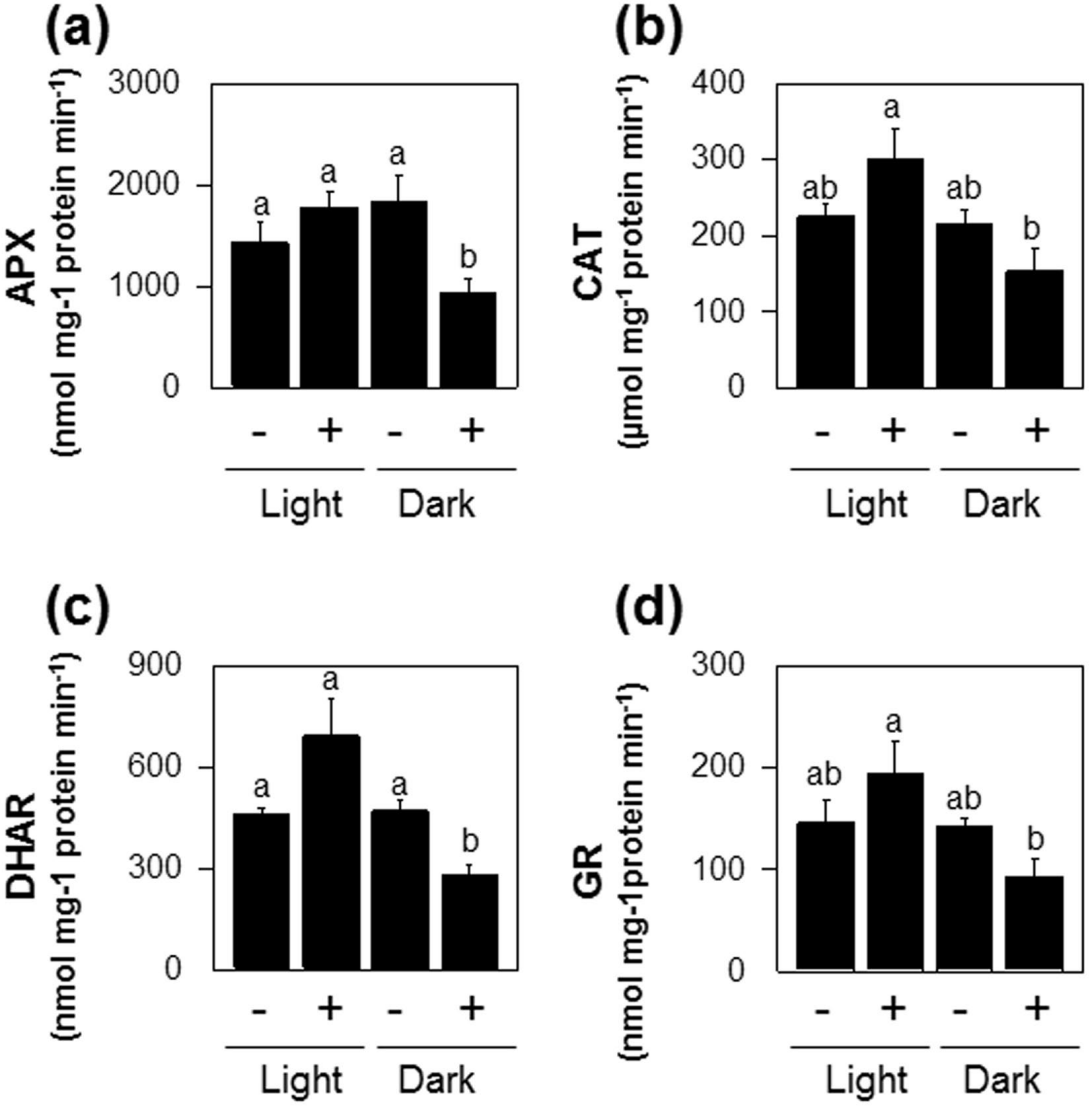
Effects of H_2_S exposure on major antioxidative enzyme in attached leaves of Col-0 under extended darkness and normal growth conditions. (**a**) APX. (**b**), CAT. (**c**), DHAR. (**d**), GR. Samples were taken from the attached leaves at 2 d of darkness and light/dark growth conditions. + and − indicate intact plants fumigated with or without 0.5 mM NaHS, respectively, during 2 d of dark incubation and light/dark growth conditions. Data are means ± SE of at least three independent samples from different plants. Letters indicates significant difference from the wild type at *P* < 0.05, using the Student’s *t* test.

To further assess the effect of the H_2_S on the ROS-antioxidant interaction, we measured the extractable activities of ascorbate peroxidase (APX), catalase (CAT), glutathione reductase (GR), and dehydroascorbate reductase (DHAR), the enzyme linking glutathione and ascorbate pools. Activities of APX and DHAR were significantly decreased in darkened plants treated with H_2_S compared with those treated with H_2_S or darkness alone, although CAT and GR activities were not greatly affected by any of the treatments (Fig. 7).

### GSNOR1 is required for H_2_S-mediated SAG expression under extended darkness

**Figure 8:**
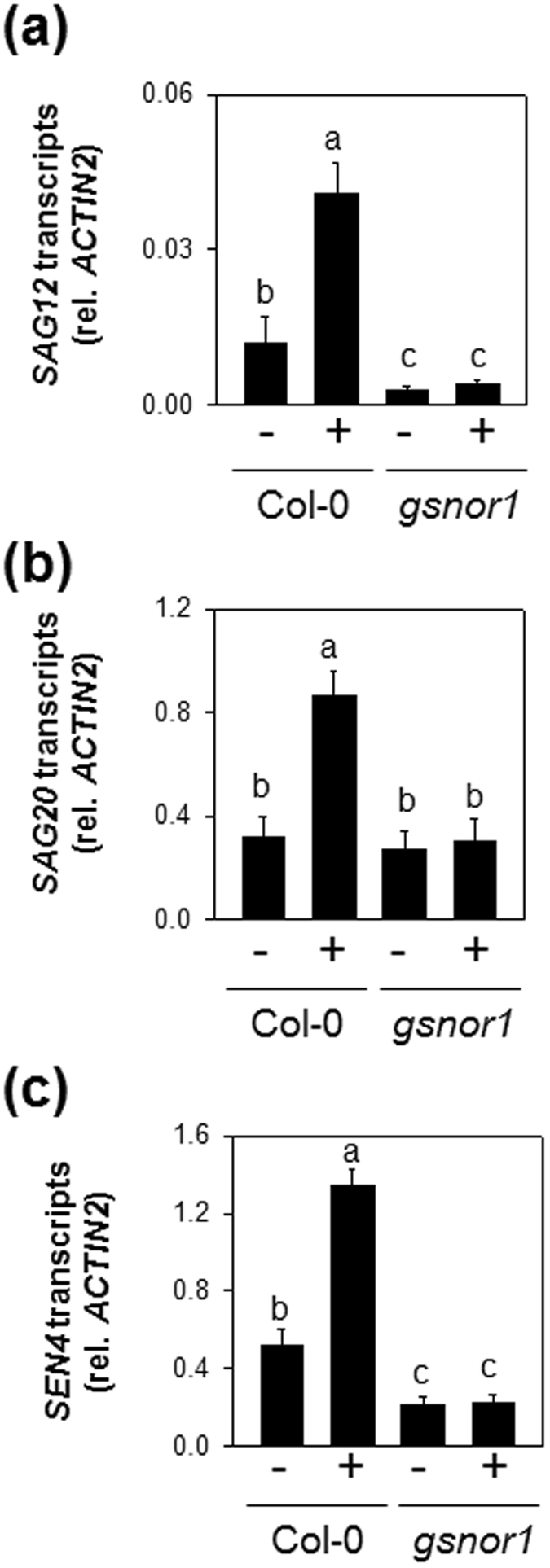
SAG expression in attached leaves of Col-0 and *gsnor1* treated with or without H_2_S under extended darkness. (**a**), *SAG12* expression. (**b**), *SAG20* expression. (**c**), *SEN4* expression. Samples were taken from the attached leaves at 4 d of darkness treatment. + and − indicate intact plants fumigated with or without 0.5 mM NaHS, respectively, during 4 d of dark incubation. Data are means ± SE of at least three independent samples from different plants. Letters indicates significant difference from the wild type at *P* < 0.05, using the Student’s *t* test.

NO has been proposed to act as a regulator of leaf senescence^58, 59^. A major route for the transmission of NO signaling is *S*-nitrosylation, a reversible post-translational modification involving the covalent addition of NO to a protein cysteine thiol to form an *S*-nitrosothiol^60^ (NO). Total cellular levels of protein *S*-nitrosylation are controlled predominantly by *S*-nitrosoglutathione reductase 1 (GSNOR1) which removes GSNO^61^. Recently, several publications reported that H_2_S interacts with NO to regulate diverse plant processes in response to adverse environmental clues^31, 35, 36^. Interestingly, many of the protein sites in Arabidopsis reported to undergo endogenous *S*-nitrosylation have also been found to undergo *S*-sulfydration. This latter reaction involves interaction of H_2_S with the thiol groups of specific proteins to form a persulfide group (R-SSH)^37, 62^. Hence, the potential role of GSNOR1 in H_2_S-regulated SAG expression was investigated in attached leaves. After 4 d of dark treatment, H_2_S-mediated induction of *SAG12*, *SAG20* and *SEN4* in H_2_S-treated Col-0 were compromised in H_2_S-treated *gsnor1* mutant (Fig. 8a–c). Taken together, these results demonstrate that GSNOR1 is involved in the H_2_S-induced expression of SAGs in attached leaves under extended darkness.

## Discussion

It is accepted that H_2_S can affect plant defense and development either by acting as a toxic molecule or as a precursor of reduced sulphur required to produce cysteine and glutathione^38^. An increasing number of reports point to regulatory functions for H_2_S in plants^6^, but the role of H_2_S in the regulation of dark-induced leaf senescence is largely unknown. In this study, a variety of approaches were exploited to understand the action of H_2_S in leaf senescence-dependent and senescence-independent processes under extended darkness. To this aim, we exploited two H_2_S donors, NaHS and GYY4137, that have been widely applied for experimental purposes in both plants and animals^13^. The concentration of H_2_S detected in plants is reported to range from 1 to 100 µM^8^. The level of gaseous H_2_S generated from 100 µM NaHS solution is close to 100 µM^22^, which is within the range of concentrations that modulate physiological processes in plants (10 to 200 µM). The concentration of fumigated H_2_S released from 0.5 mM NaHS (200 mL) in 3 L sealed containers is around 33 µM. Therefore, most experiments in this work were conducted using this physiologically relevant concentration range.

### H_2_S represses chlorophyll breakdown via a mechanism that is independent of anti-senescence processes

Darkness is often used to induce rapid and synchronous senescence in detached leaves, and chlorophyll catabolism is an integral process of leaf senescence^50^. In the present study, our findings demonstrate that H_2_S has a negative effect on chlorophyll degradation under extended darkness, but that this effect is uncoupled from the expression of SAGs (Fig. 1). Furthermore, like in the stay-green mutant *pao1*, our results show that the presence of H_2_S results in the accumulation of pheide *a* during dark incubation (Fig. 2c), further supporting the existence of a feedback mechanism that limits metabolism of chlorophyll in H_2_S-treated detached leaves or in mutants that are unable to degrade chlorophyll beyond pheide *a* ^53^. Hence, H_2_S probably inhibits chlorophyll breakdown at the level of pheide *a* under extended darkness. Additionally, the accumulation of pheide *a* is reported to be responsible for the cell death phenotype on leaves in a light-dependent way^52^. Consistent with this notion, cell death or rapid bleaching after transfer to light is apparently observed in both detached and attached leaves treated with H_2_S (Figs 2 and 3). The results presented here imply (1) that pheide *a* metabolism is important in linking H_2_S to a downstream “stay-green” phenotype under extended darkness, (2) that pheide *a* is required for the cell death reaction observed in H_2_S-treated leaves shifted from extended darkness to light, (3) and that H_2_S suppresses chlorophyll degradation of detached leaves through regulating unidentified dark-dependent reactions rather than modulating anti-senescence processes. In addition, formation of the colorless primary fluorescent chl catabolite (pFCC) from RCC *a* is responsible for the loss of green pigment in chlorophyll breakdown, while RCC accumulation causes leaf cell death^54^. Therefore it would be interesting to understand if RCC mediates H_2_S-associated responses.

### H_2_S potentiates dark-induced expression of SAGs in attached leaves

Recently, Álvarez *et al*. (2012) reported that mutation of *DES1* led to a 30% reduction in endogenous sulfide and early age-associated senescence as evidenced at the cellular and transcriptional levels^45^. DES1 deficiency promoted accumulation of *de novo* senescence-associated vacuoles and the expression of *SAG12* and *NAP* ^9, 45^. This is markedly different from what we observed. Our results show that exogenously applied H_2_S promotes higher transcript levels of several SAGs compared with H_2_S-untreated attached leaves (Fig. 4a–c). Moreover, *des1* mutants and two independent *DES1* transgenic lines show, respectively, decreased or enhanced expression of SAGs during extended darkness (Figs 4b–d and 5). This apparent discrepancy appears to be the cause of the difference between age-triggered senescence and dark-induced senescence. It is possible that the SA pathway is specifically involved in age-dependent leaf senescence^50^. SA is not only a key plant hormone mediating the plant response to pathogens but also functions in leaf senescence. Higher SA levels have been reported in senescing Arabidopsis leaves, and this observation is accompanied by the induction of genes such as *SAG12* ^63^. Consistent with this possibility, the levels of SA and SA-responsive defense markers such as *PR1* are significantly increased in the *des1* mutant, correlating with the up-regulation of several SAG genes including *SAG12* and *SAG21* during age-related senescence^9, 45, 64^. However, we observed no difference in *PR1* expression in attached leaves in the presence or absence of H_2_S during extended darkness (Fig. 5d).

Elevated levels of H_2_O_2_, either through enhanced H_2_O_2_ generation or down-regulation of antioxidant levels, could promote senescence^47, 65^. In agreement, increases in SAG expression in H_2_S-treated attached leaves under extended darkness were accompanied by increased H_2_O_2_ and decreased GSH (Figs 4 and 6). Failure of H_2_S-treated detached leaves to further enhance the expression levels of SAGs was perhaps due to decreased accumulation of oxidants (Supplementary Fig. S2c,d). Thus, it would be interesting to investigate further the role of redox regulation in H_2_S-mediated senescence processes.

### GRSNOR1 is required for H_2_S-mediated expression of SAGs in attached leaves under extended darkness through modulating SNO level

Although *S*-sulfhydration has been proposed as a likely mechanism of H_2_S signaling in mammalian systems, evidence for this process has only been very recently reported in plants^37^. One hundred and six *S*-sulfhydrated proteins were identified in Arabidopspis, many of which also underwent *S*-nitrosylation^62^. Moreover, recent work found that H_2_S treatment can suppress the accumulation of SNO by enhancing GSNOR enzyme activity^66^. These reports are consistent with several lines of evidence that point to an interaction between H_2_S and NO in plant growth and defenses^17^. *S*-nitrosylation typically inhibits protein function. In contrast, *S*-sulfhydration can activate enzymatic activities. For instance, *S*-nitrosylation negatively regulates the activities of a cytosolic ascorbate peroxidase, APX1^67^, and a cytosolic glyceraldehyde-3-phosphate dehydrogenase, GAPC1^68^, in plants. Both of these enzymes can also be *S*-sulfhydrated by H_2_S, which increases their activities^37^. If H_2_S regulates dark-induced senescence through *S*-nitrosylation mechanisms, enhanced SNO levels may attenuate the effects of H_2_S. This is a possible explanation of why H_2_S-mediated induction of SAG expression was compromised by the *gsnor1* mutation (Fig. 8). Thus, appropriate modulation of SNO levels by GSNOR1 is crucial to H_2_S-regulated SAG expression triggered in darkened attached leaves. Although studies on the downstream targets of H_2_S signal functioning in plant responses to stress are still quite limited, the effects reported here clearly point to interplay between H_2_S and NO in post-translationally determining the status of protein thiols^37, 62^.

## Methods

### Plant material and growth conditions

*Arabidopsis thaliana* wild-type Columbia-0 (Col-0), *atg2* (SALK_076727), *atg5* (SAIL_129B07), *atg18a* (GABI_651D08), *cad2* ^69^, *des1* (SALK_103855) and *gsnor1* (GABI_315D11) lines were used in this work. Seeds were incubated for 2 d at 4 °C and then sown in soil. Plants were grown in soil in a controlled-environment growth chamber in a 16 h photoperiod and an irradiance of 120 μmol m^−2^ s^−1^ at leaf level, 22 °C day/20 °C night, 65% humidity and given nutrient solution twice per week. Samples were rapidly frozen in liquid nitrogen and stored at −80 °C until analysis. Unless otherwise stated, data are means SE of at least three independent samples from different plants.

### Hydrogen sulfide fumigation and dark treatment

Solutions of sodium hydrosulfide (NaHS•3H_2_O) were used as one of the hydrogen sulfide (H_2_S) donors. To examine the dose effect of H_2_S on leaf yellowing, aqueous solutions (200 mL) of 0 (control), 0.01, 0.1, 0.5, 1 or 2 mM NaHS were prepared, from which H_2_S gas was released in a sealed glass desiccator (volume 3 L). For SAG expression analysis, excised leaves from 3- to 4-week-old plants incubated on wet filter paper or attached leaves from either 10-day-old or 3-week-old seedlings were kept in the presence or absence of H_2_S released from 0.5 mM NaHS solution in darkness for several days. For cell death assay, detached and attached leaves of 3- to 4-week-old plants were kept in darkness in combination with 0.5 mM NaHS for 2 or more days and then transfer to 16 h/8 h photoperiod conditions. The NaHS solutions were renewed each two days and treated leaves were collected at designated time intervals for analyses.

To confirm the effects of gaseous H_2_S on the senescence of Arabidopsis leaves, 0.1 mM Morpholin-4-ium 4-methoxyphenyl (morpholino) phosphinodithionate (GYY4137) was used as a second H_2_S donor, while 0.1 mM hypotaurine (HT) was used as an H_2_S scavenger.

### Thiol treatment

To study the effect of cysteine and glutathione on dark-triggered leaf senescence, detached leaves from 3- to 4-week-old plants were placed in petri dishes containing 3 mL solution of 0.1 mM cysteine or 0.1 mM glutathione under extended darkness.

### Generation of *DES1* transgenic plants

*DES1* cDNA was amplified from Arabidopsis with primer pairs of DES1-F2/DES1-R2 by RT-PCR. After verifying the sequence fidelity by sequencing, these products were cloned into the *XbaI* and *XhoI* sites of pBI121 under the control of 35 S promoter. The 35 S::*DES*1 construct was introduced into the *Agrobacterium tumefaciens* GV3101 strain, which was then used to transform Col-0 using the flower infiltration method. Two independent lines overexpressing *DES1* were identified and characterized for further analyses. All transgenic lines used in this study were T3 homozygous plants.

### RT-qPCR analyses

Total RNA was extracted from the designated tissues using Trizol (Invitrogen). 2 μg of total RNA was used for the synthesis of the first-strand cDNA using the All-in-One cDNA Synthesis Super Mix and oligo dT as primers (Biotool). Quantitative PCR was performed in a StepOnePlusTM Real-Time PCR System (Applied Biosystems) using 2x SYBR Green qPCR Master Mix (High ROX) (Biotool). Transcript levels of target genes were normalized to that of the housekeeping gene *ACTIN2* (AT3G18780) using the equation of 2^−ΔCT^, where CT is the threshold cycle for each gene in the sample. The primers used are listed in Supplementary Table S1.

### Measurement of DES activity and H_2_S content

L-Cysteine desulfhydrase (DES; EC 4.4.1.1) activity was determined according to the method of Riemenschneider *et al*.^12^. This method is based on the measurement of catalytic release of sulfide from cysteine. The soluble proteins were extracted by adding 1 mL of 20 mM Tris-HCl (pH 8.0), and centrifuged at 15,000 g for 15 min at 4 °C. The reaction mixture (1 mL) consisted of 2.5 mM dithiothreitol (DTT), 0.8 mM L-Cysteine, 100 mM Tris-HCl (pH 9.0), and enzyme extract. The reaction was initiated by the addition of L-Cysteine. The reaction mixture was incubated at 37 °C for 15 min, and then the reaction was terminated by the addition of 0.1 mL of 30 mM FeCl_3_ dissolved in 1.2 N HCl and 0.1 mL 20 mM N, N-dimethyl-p-phenylenediaminedihy drochloride dissolved in 7.2 N HCl. The formation of methylene blue was determined at 670 nm. DES enzymatic activity was calculated using a standard curve prepared with NaHS. D-Cysteine desulfhydrase activity was determined in the same way, but D-Cysteine was used instead of L-Cysteine.

The determination of H_2_S was carried out according to the method of Singh *et al*.^70^. 0.5 g plant leaves was ground into fine powder with a mortar and pestle under liquid nitrogen and then homogenized in 1 ml of the following extraction buffer: 20 mM Tris-HCl buffer (pH 8.0), 10 mM EDTA, 20 mM Zn(OAc)_2_. The homogenate was centrifuged at 15,000 g for 15 min at 4 °C. The reaction mixture (2 mL) consisted of 0.1 mL supernatant, 1.88 mL extraction buffer and 0.02 mL of 20 mM 5,5′-dithiobis(2-nitrobenzoic acid). The reaction mixture was incubated at room temperature for 2 min and absorbance was recorded at 412 nm. The level of H_2_S was calculated according to a standard curve of NaHS.

### Analyses of chlorophyll and green catabolites

For spectrophotometric determination of chlorophyll level, chlorophyll was extracted from leaf tissue by homogenization in liquid nitrogen and subsequent threefold extraction into 80% (v/v) acetone containing 1 mM KOH. After centrifugation (10 min, 12,000 g), supernatants were combined and used for analysis. The absorbance of the supernatant was read at 663 and 645 nm, and the amount of total chlorophyll (μg/mL) was calculated as 8.02 × A_663_ + 20.2 × A_645_^71^.

For HPLC analyses of green chlorophyll *a* catabolites (pheophytin *a* and pheide *a*), liquid nitrogen-homogenized tissue was extracted in 10% (v/v) 0.2 M Tris-HCl (pH 8.0) in acetone, and incubated at −20 °C for 2 h in the dark. After removal of insoluble material by centrifugation (10 min, 12,000 g), supernatants were analyzed by reverse-phase HPLC as previously described^52^.

### Ion Leakage

For ion conductivity analysis, detached leaves of 3- to 4 weeks plant were incubated in the presence or absence of H_2_S released from 0.5 mM NaHS solution in the dark for 2 d. Eight leaves for each treatment were then soaked in 10 ml of distilled water in a test tube. After re-exposure to light (120 μmol m^−2^ s^−1^) for up to 12 h, ion leakage as a measure of cellular damage was determined by measuring the conductivity of the solution with a FiveGo F3 meter (Mettler Toledo).

### Antioxidant enzyme assays metabolite, and H_2_O_2_ analyses

Extractable enzyme activities were measured as described in Noctor *et al*.^72^. Oxidized and reduced forms of glutathione and ascorbate were measured by plate-reader assay as described in Queval and Noctor^57^. H_2_O_2_ content was determined by the method of titanium oxidation with hydroperoxide-titanium complex formed^73^.

### Statistical analysis

The statistical analysis of data was based on Student’s *t* tests. Calculations were performed on a minimum of three independent datasets, assuming two samples equal variance and a two-tailed distribution. Unless stated otherwise, significant difference was assessed using multiple pair wise *t* test comparisons at *P* < 0.05.

## Electronic supplementary material

Supplementary Information
