# Supplementary material for: Functional analysis of the role of hydrogen sulfide in the regulation of dark-induced leaf senescence in Arabidopsis
Source: Sci Rep. 2017 Jun 1;7:2615. doi: 10.1038/s41598-017-02872-0 (PMC5454012; doi:10.1038/s41598-017-02872-0)
Supplement: Supplementary file 1 — Supplementary Information [file 41598_2017_2872_MOESM1_ESM.pdf]

Supplementary information

Functional analysis of the role of hydrogen sulfide in the regulation of dark-induced leaf senescence in Arabidopsis

Bo Wei[a], Wei Zhang[a], Jin Chao[a], Tianru Zhang[a], Tingting Zhao[a], Graham Noctor[b], Yongsheng Liu[a,*] and Yi Han[a,*]

[a] School of Food Science and Engineering, Hefei University of Technology, Hefei, Anhui, 230009 China.

[b] Institute of Plant Sciences Paris Saclay, Université Paris-Sud, Centre National de la Recherche Scientifique, Institut National de la Recherche Agronomique, Université Evry, Paris Diderot, Sorbonne Paris-Cité, Université Paris-Saclay, 91405 Orsay, France

*To whom correspondence should be addressed E-mail: yi.han@hfut.edu.cn and liuyongsheng1122@hfut.edu.cn

Supplementary Table S1. Sequences of primers used in this work.

Locus	Oligonucleotide	Sequence (5' → 3')	Restriction enzyme	Purpose
AT1G69490	NAP-F	CGAAGCAGAGAGAAGAACTGAA		RT-qPCR
	NAP-R	CAAATGAGCCAGCGAACAC		
AT4G22920	SGR1-F	GGGAAAATGTCGCTTCACG		RT-qPCR
	SGR1-R	AGCCTTCAACACCACAGGTAG		
AT5G13800	PPH-F	AATCCCCAACGCTCCATACT		RT-qPCR
	PPH-R	CTTCAAAACCACCAGACTCCA		
AT3G44884	PAO-F	CCCAGGCAGACCGTTTTGT		RT-qPCR
	PAO-R	TGACTCTTACCATGCCGTCTGA		
AT4G13250	NYC1-F	GCAGAGAACAGGACGAGGTT		RT-qPCR
	NYC1-R	CGCAAACAACAGAAAGAGAGAA		
AT5G04900	NOL-F	CCGACTTACATCCGTTTCCTAA		RT-qPCR
	NOL-R	CTGTTCTTCCTTGCTCCCAAC		
AT1G19670	CLH1-F	CCCGTCGTTTTATTCTTCCA		RT-qPCR
	CLH1-R	AGCATCGTCCACTTCCACTT		
AT5G43860	CLH2-F	ACAAAAGGGATTAGAGGGAAGAG		RT-qPCR
	CLH2-R	GATACAACAAGTCCACCAACGA		
AT4G37000	RCCR-F	CGCCGAAAATTTATGGAGTT		RT-qPCR
	RCCR-R	AGGGAAGGAGTTGTGATTGG		
AT5G45890	SAG12-F	GGCGTTTTCAGCGGTTGCGG		RT-qPCR
	SAG12-R	CCGCCTTCGCAGCCAAAATCG		
AT3G10985	SAG20-F	TCGGTAACGTTGTTGCTGGA		RT-qPCR
	SAG20-R	ACCAAACTCTTTCAAATCGCCA		
AT4G30270	SEN4-F	GACTCTTCTCGTGGCGGCGT		RT-qPCR
	SEN4-R	CCCACGGCCATTTCCCCAAGC		
AT3G18780	ACTIN2-F1	CTGTACGGTAACATTGTGCTCAG		RT-qPCR
	ACTIN2-R1	CCGATCCAGACACTGTACTTCC		
AT5G28030	DES1-F1	GGATTGGTTTAGCCAGCATC		RT-PCR
	DES1-R1	CACCAGAGCCTATTCCTTGG		
AT3G18780	ACTIN2-F2	CTGTGCCAATCTACGAGGGT		RT-PCR
	ACTIN2-R2	TGCTCATACGGTCAGCGATA		
AT5G28030	DES1-F2	TCTAGA ATGGAAGACCGCGTCTTGATC		over expression
	DES1-R2	CTCGAG CTACTCATTCAACTGGCAAATTC		
AT4G23100	CAD2-F	AGCATTTCACTTGAACCTGG	*BsI1*	Genotyping
	CAD2-R	TCCTTGTCAGTGTCTGTCC		
AT3G19190	ATG2-F	GCACTTTCCATCAGCTACTCG		Genotyping
	ATG2-R	CATTCGAGGTTCTGGCCTAAC		
AT5G17290	ATG5-F	GCTAATTGCACAAAGCTTACCTC		Genotyping
	ATG5-R	TGATATGCCTAACATCGTCCAC		
AT3G62770	ATG18A-F	AACCCTAATCCCGATTCCAC		Genotyping
	ATG18A-R	ACATGGACCGTTCCTTTGTC		
AT5G43940	gsnor1-F	TTCGAATGACACGAAAATCG		Genotyping
	gsnor1-R	TAAGCGTCGGTGTGACAAAG		

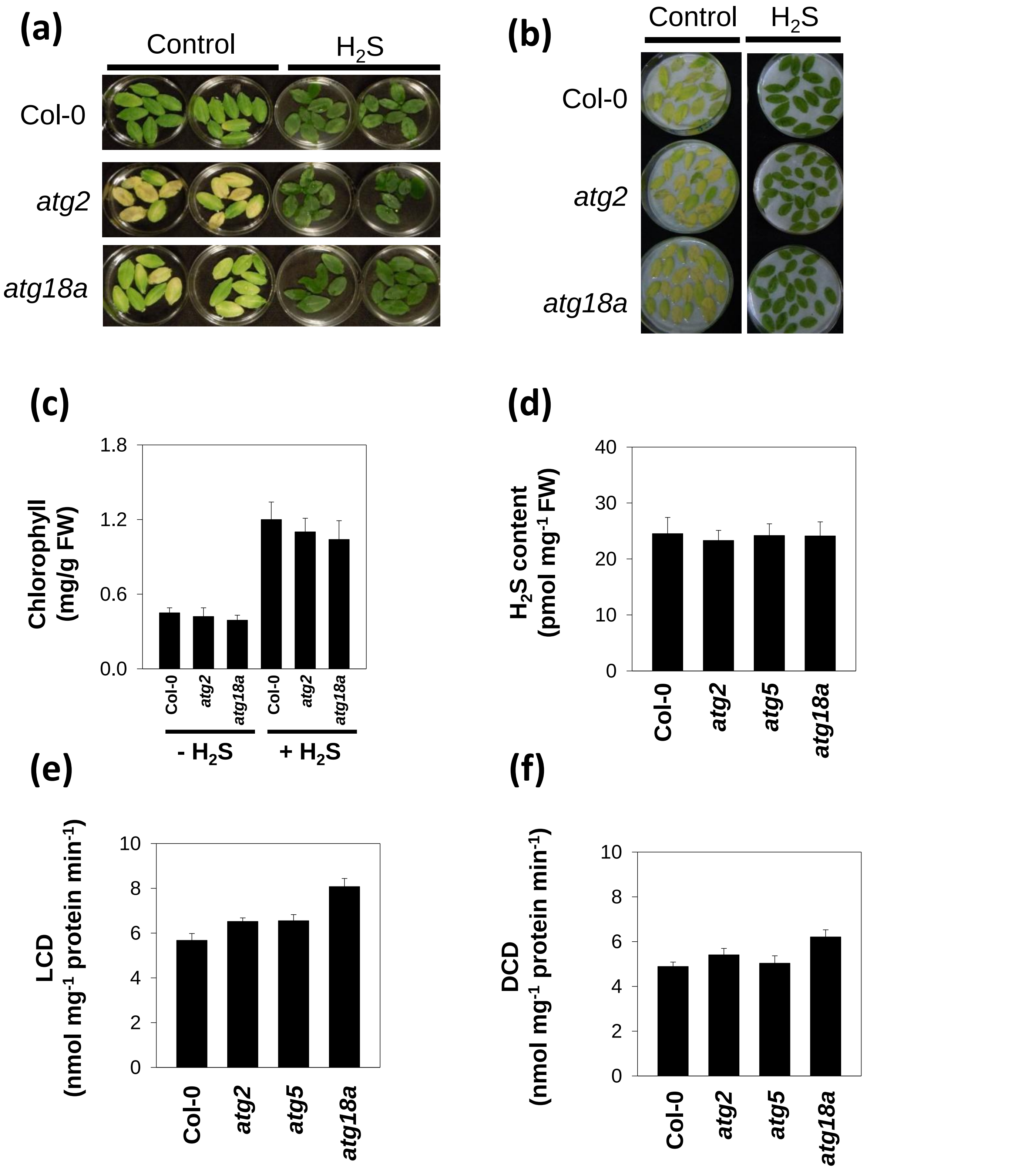

Supplementary Figure S1. Analyses of the interplay between autophagy and H_2S in the regulation of chlorophyll breakdown during extended darkness. (a) and (b), Phenotype of *atg2* and *atg18a* detached leaves treated with or without 0.5 mM H_2S, respectively, under extended darkness for 2 d or 4 d. (c), Effect of H_2S on chlorophyll contents of autophagy deficient mutants (*atg2* and *atg18a*) under extended darkness for 4 d. + and - indicate detached leaves of wild-type and autophagy mutants fumigated with or without 0.5 mM NaHS respectively. (d) to (f), H_2S content, LCD and DCD activities in *atg2*, *atg5*, and *atg18a* mutant. Data are means ± SE of at least three independent samples from different plants.

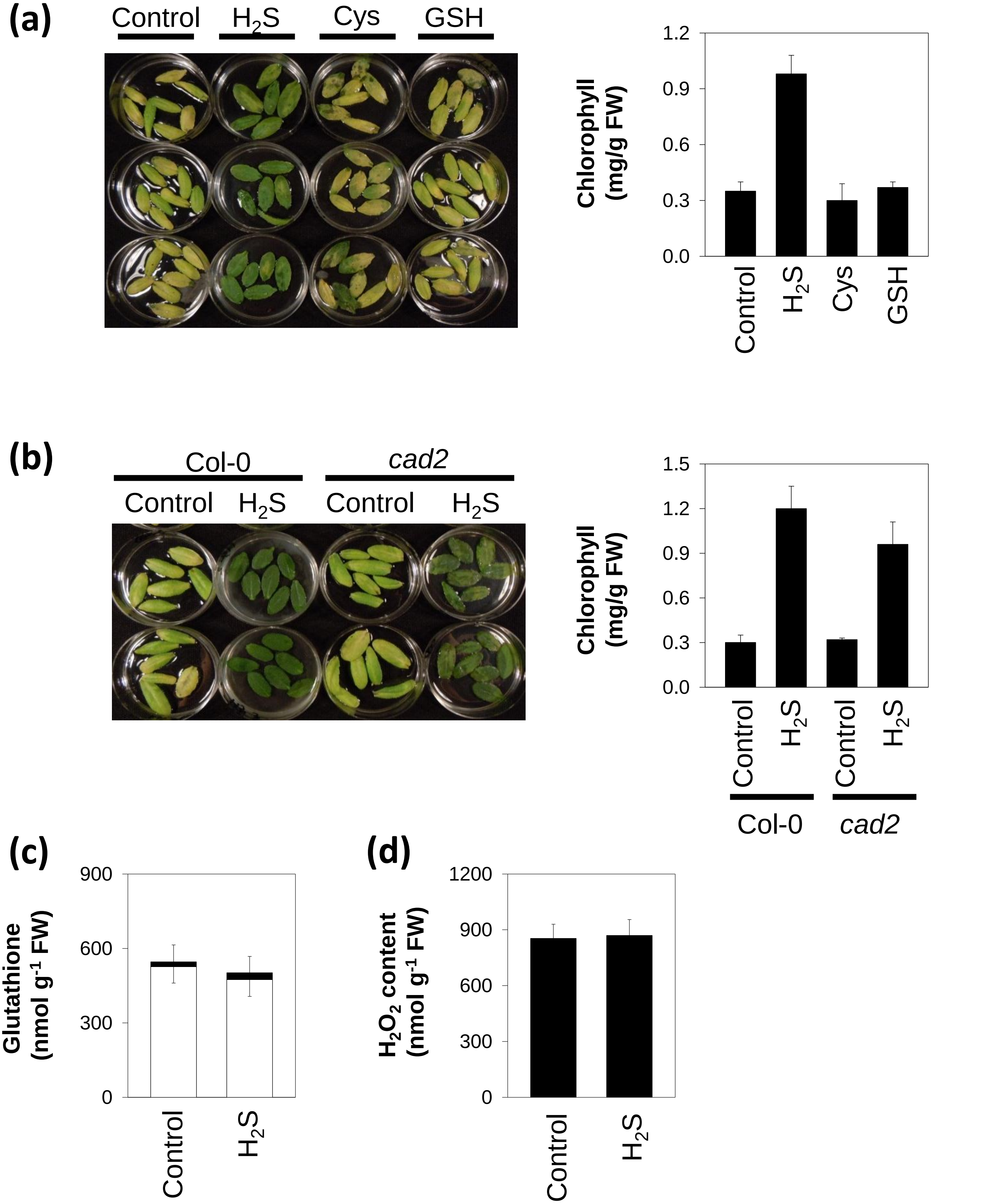

Supplementary Figure S2. Effects of H_2S-linked components on chlorophyll degradation, glutathione and H_2O_2 level, during extended darkness. (a), effects of exogenous applied cysteine with 0.1 mM or glutathione with 0.1 mM on dark-induced leaf color and chlorophyll breakdown and in detached leaves. (b), phenotype of detached leaves and chlorophyll contents in Col-0 and *cad2* treated with or without H_2S under extended darkness for 4 d. (c), glutathione level in detached leaves of wild type plants treated with or without H_2S under extended darkness for 4 d. Reduce glutathione (white bars) and oxidized form (black bars). (d), H_2O_2 content. Samples were taken from the detached leaves at 4 d of darkness in the presence or absence of H_2S.

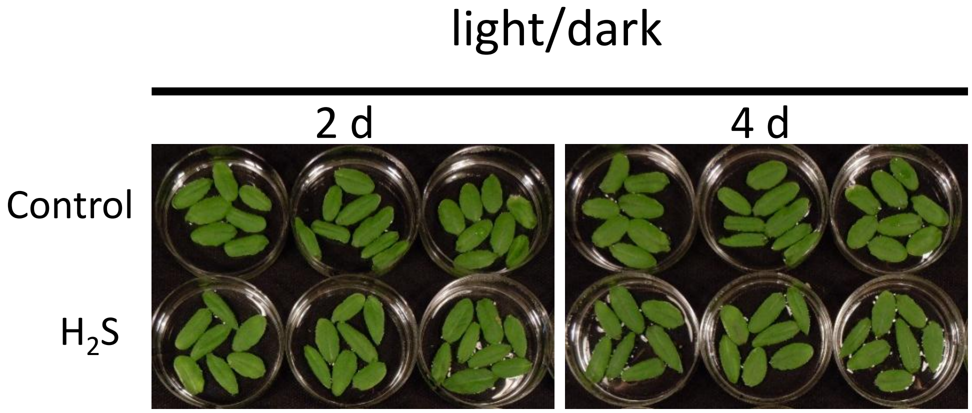

Supplementary Figure S3. Effects of H_2S exposure on phenotype of detached leave of Col-0 under regular light/dark conditions.

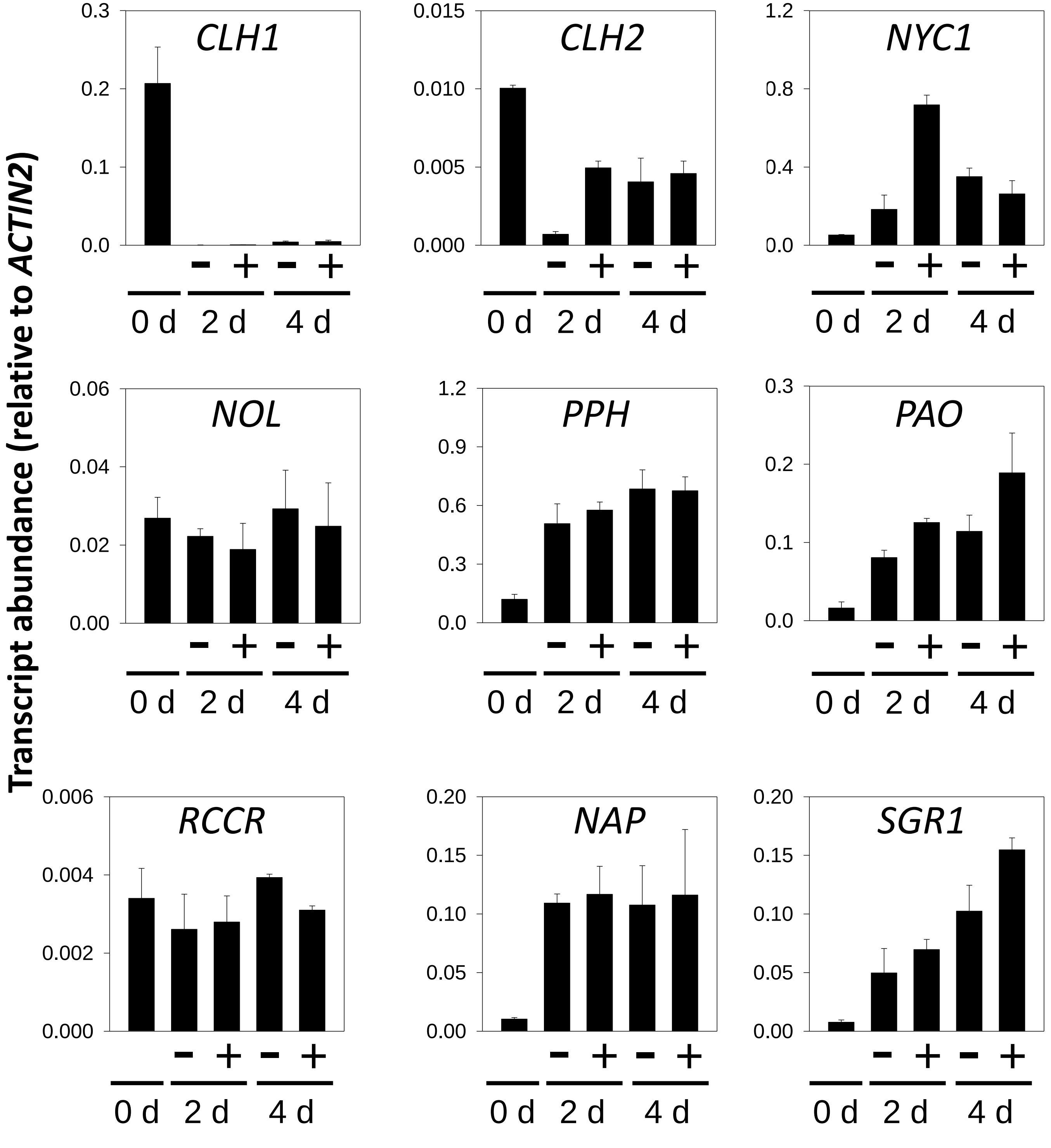

Supplementary Figure S4. Relative transcript levels of chlorophyll catabolic genes in H_2S-treated detached leaves during extended darkness. *CLH1* (*CHLOROPHYLLASE 1*), *CLH2* (*CHLOROPHYLLASE 2*), *NYC1* (*NON-YELLOW COLORING 1*), *NOL* (*NYC1-LIKE*), *PPH* (*PHEOPHYTINASE*), *PAO* (*PHEOPHORBIDE A OXYGENASE*), *RCCR* (*RED CHLOROPHYLL CATABOLITE REDUCTASE*), *NAP* (*NAC-LIKE, ACTIVATED BY AP3/PI*), *SGR1* (*STAY-GREEN1*). + and - indicate entire plants treated with or without 0.5 mM NaHS, respectively, during dark incubation. Data are means ± SE of at least three independent samples from different plants.

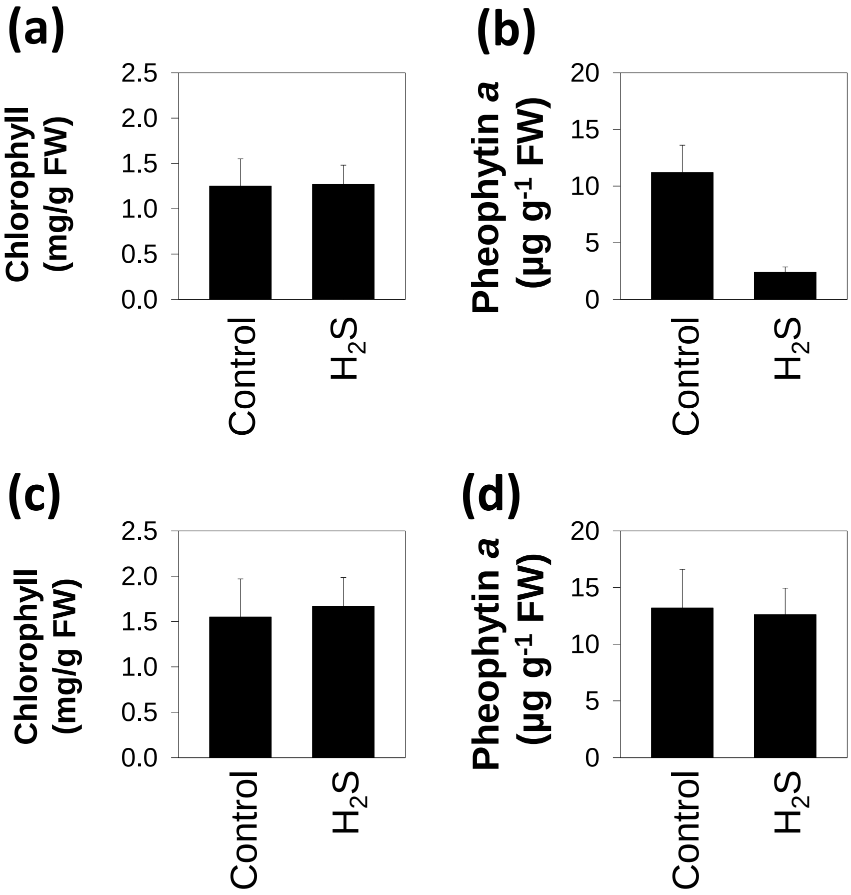

Supplementary Figure S5. Effects of H_2S exposure on the levels of chlorophyll and pheophytin *a* of attached leaves of Col-0 under prolonged darkness and regular growth conditions. (a), Chlorophyll, samples were taken from attached leaves of Col-0 at 2 d of darkness treatment. (b) Pheophytin *a*, samples were taken from attached leaves of Col-0 at 1 d regular growth conditions after transfer from 2 d of dark/H_2S treatment. (c), Chlorophyll content. (d), pheophytin *a* level. samples were taken from attached leaves of Col-0 at 3 d of regular growth conditions. Wild-type plants were fumigated with or without 0.5 mM NaHS respectively. Data are means $\pm$ SE of at least three independent samples from different plants.

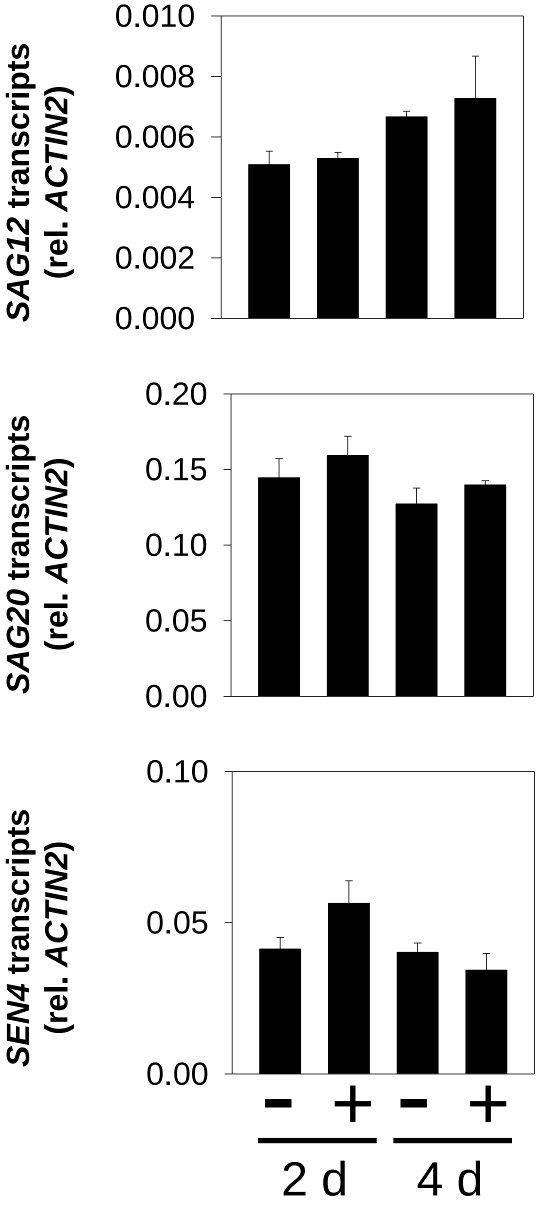

Supplementary Figure S6. Effects of H_2S exposure on SAG expression in attached leaves under normal growth conditions. + and - indicate entire plants fumigated with or without 0.5 mM NaHS, respectively, during growth. Data are means ± SE of at least three independent samples from different plants.

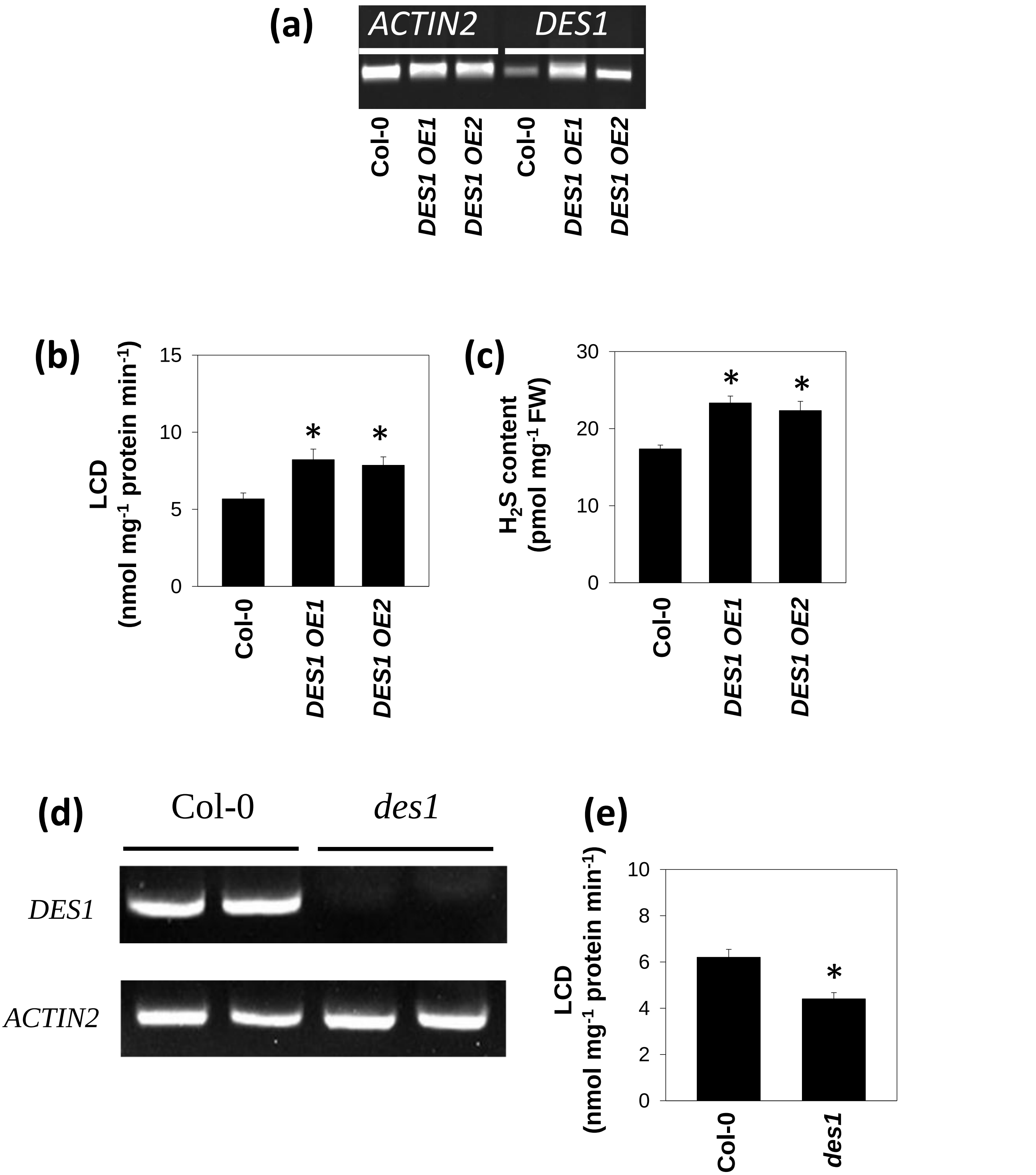

Supplementary Figure S7. Identification and characterization of *DES1* transgenic lines and *des1* mutant. RT-PCR for *DES1* expression (a), LCD activity (b) and DCD activity (c) are determined in two independent *DES1* transgenic lines. RT-PCR for *DES1* expression and LCD activity of *des1* mutant are shown in (d) and (e) respectively. Data are means ± SE of at least three independent samples from different plants. Asterisks indicate significant difference from the wild type at the same time point at $P < 0.05$, using the Student's *t* test.

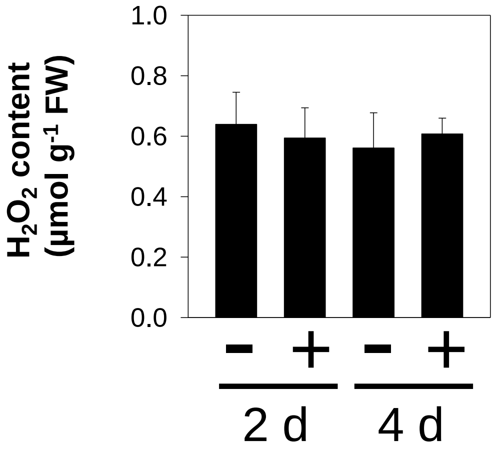

Supplementary Figure S8. Effects of H_2S exposure on H_2O_2 content in attached leaves of Col-0 under normal growth conditions. + and - indicate entire plants fumigated with or without or with 0.5 mM NaHS, respectively, during growth. Data are means ± SE of at least three independent samples from different plants.

(a)

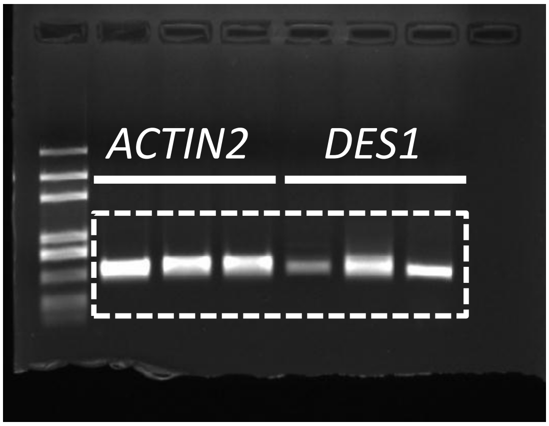

(b)

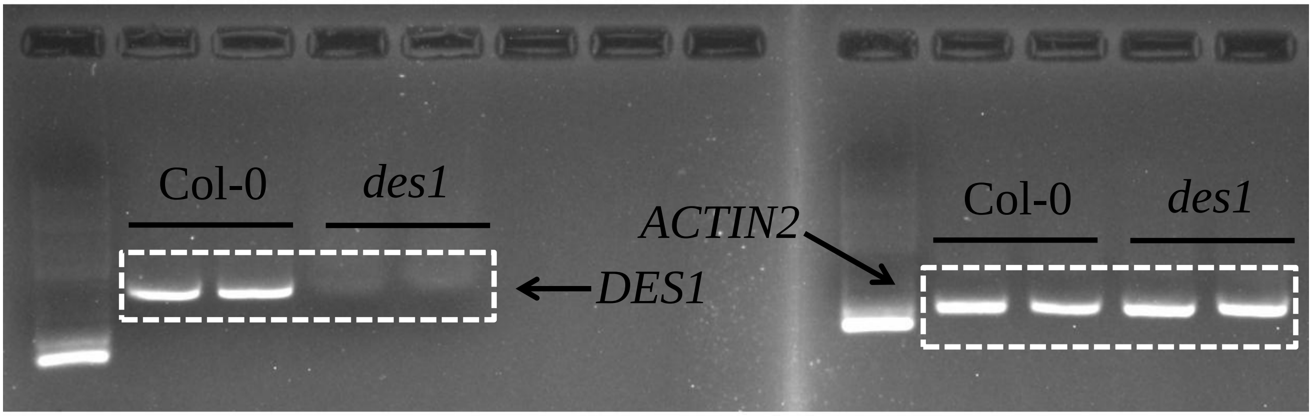

Supplementary Figure S9. Full-length image of supplementary Figure S7a (a) and supplementary Figure S7 d (b) . White dotted lines indicate the cropping locations.
